# Supplementary figures and images for: Extent of integration of nutrition assessment counselling and support interventions in the health system and respective drivers: A case of Tororo district, Uganda
Source: PLoS One. 2023 Dec 21;18(12):e0289389. doi: 10.1371/journal.pone.0289389 (PMC10735038; doi:10.1371/journal.pone.0289389)

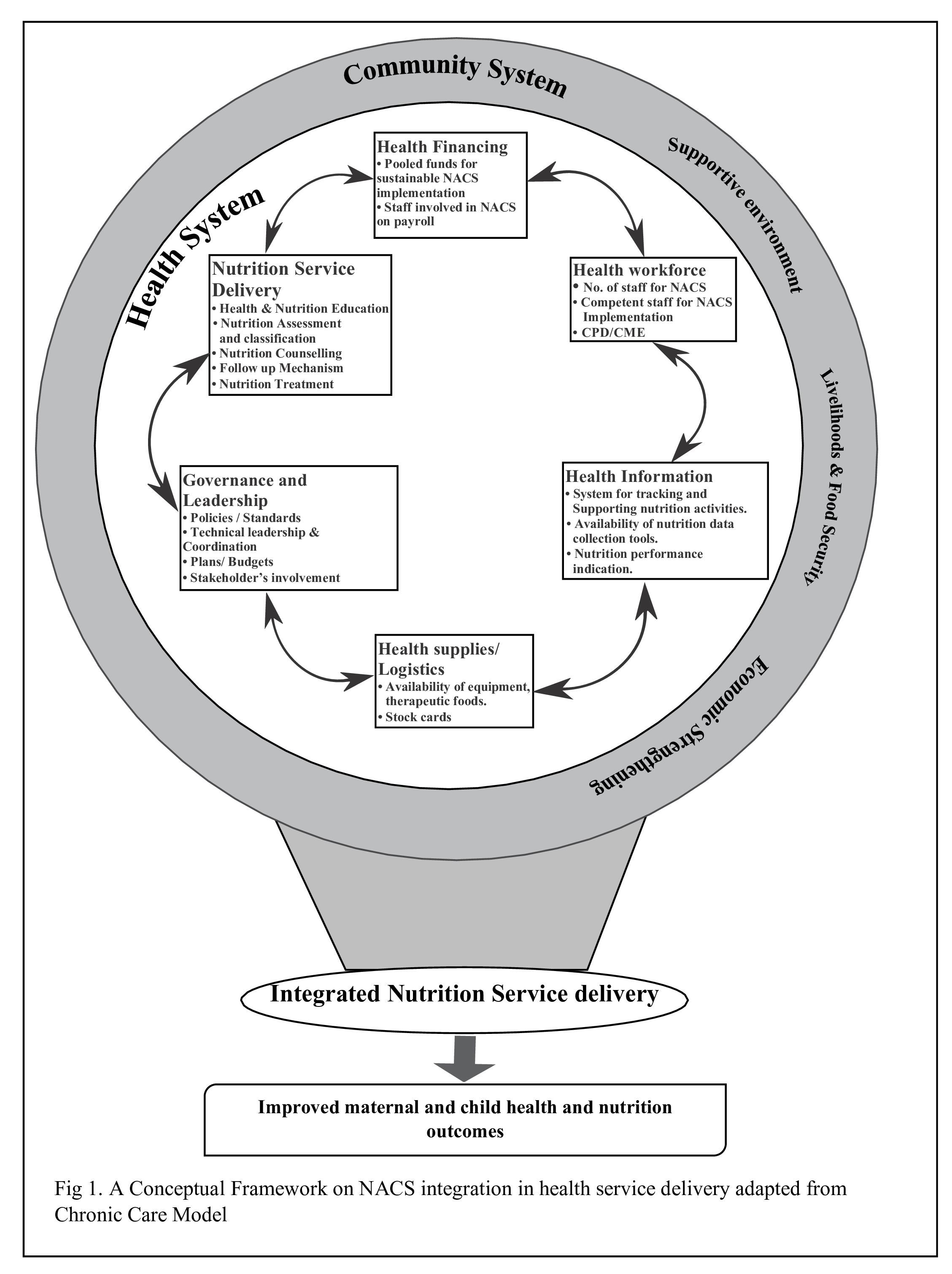

Supplement: S1 Fig — (TIFF) [file pone.0289389.s003.tiff]

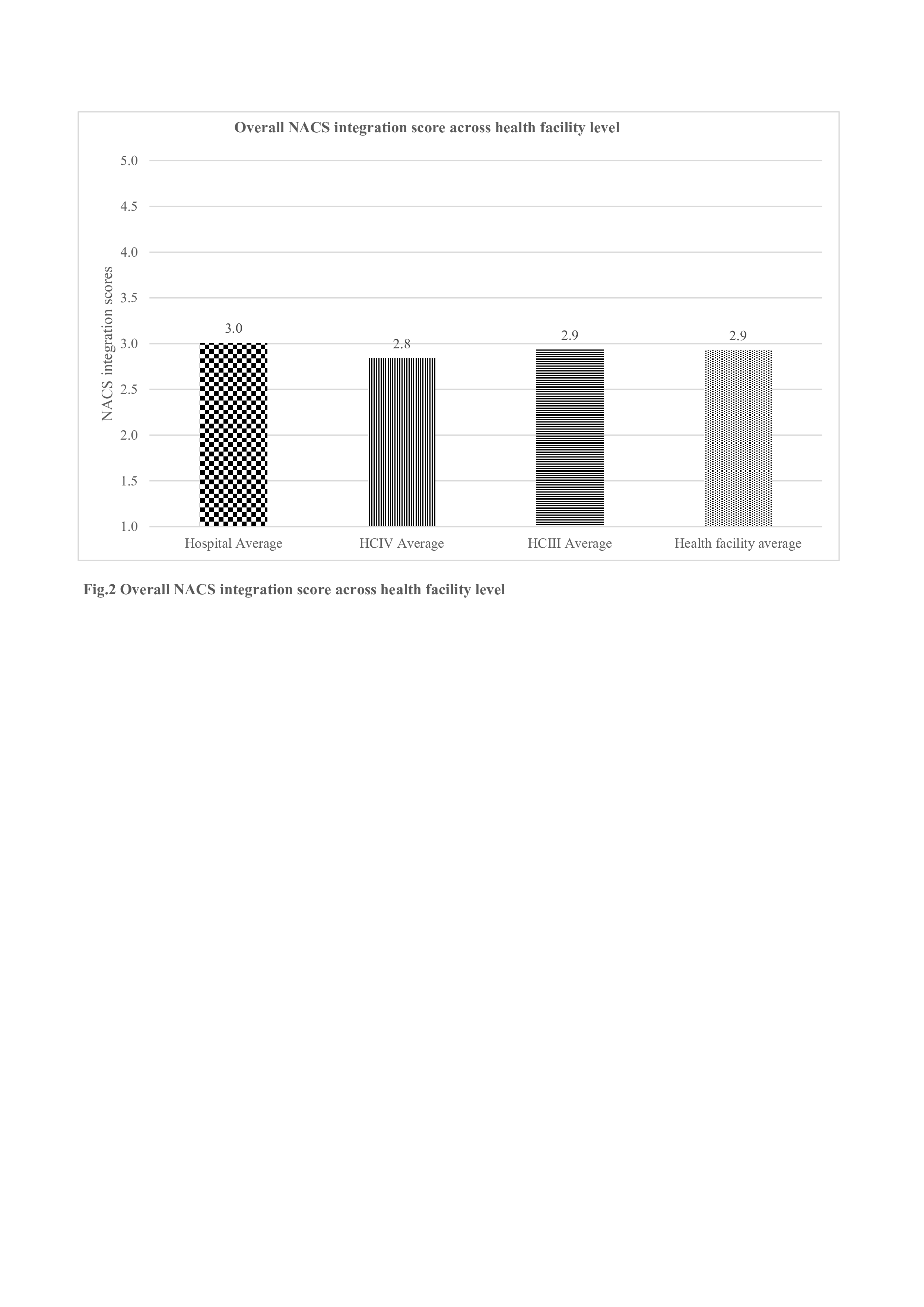

Supplement: S2 Fig — (TIFF) [file pone.0289389.s004.tiff]

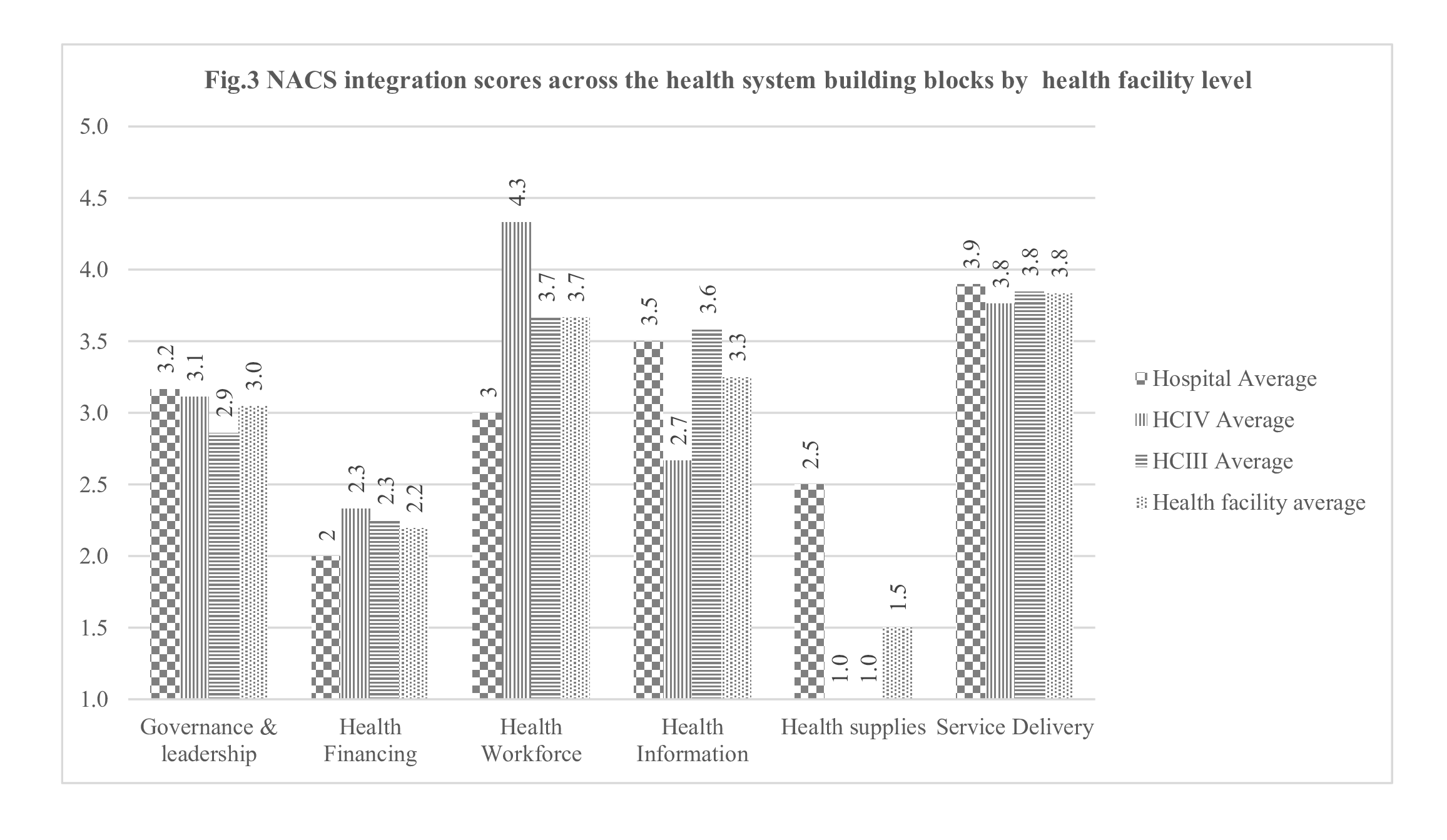

Supplement: S3 Fig — (TIFF) [file pone.0289389.s005.tiff]

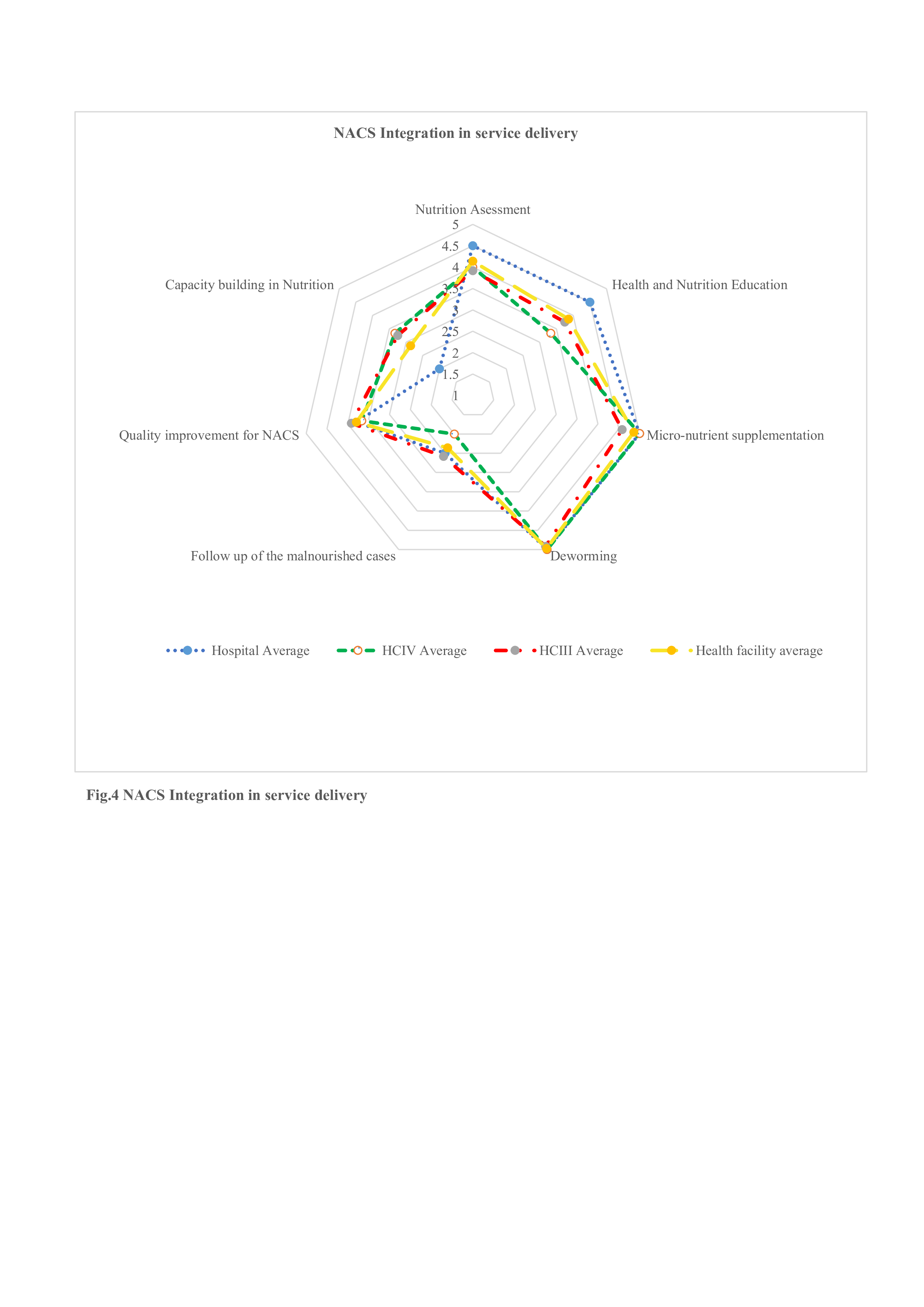

Supplement: S4 Fig — (TIFF) [file pone.0289389.s006.tiff]

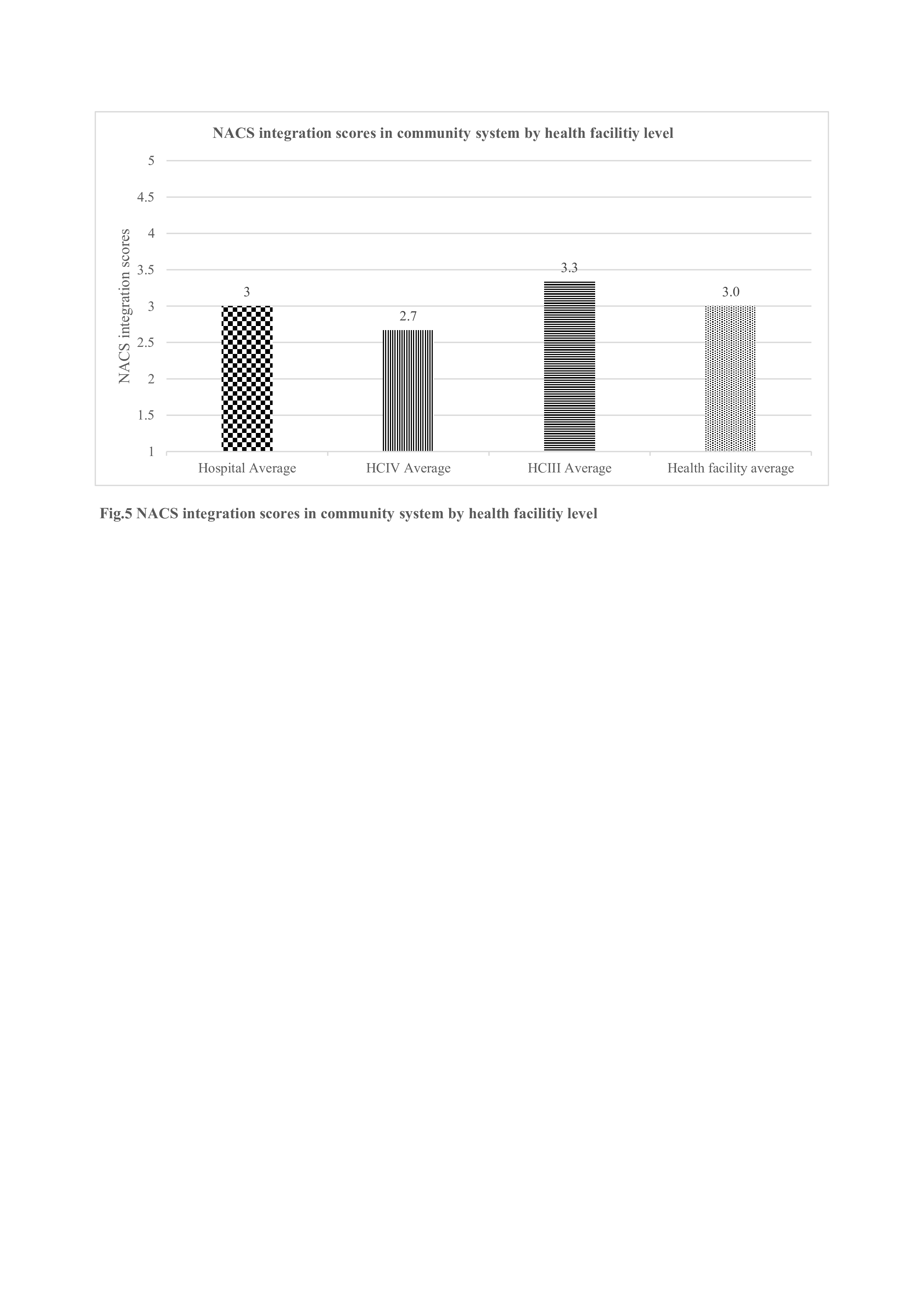

Supplement: S5 Fig — (TIFF) [file pone.0289389.s007.tiff]
